# Optimization of ethylene glycol production from (d)-xylose via a synthetic pathway implemented in *Escherichia coli*

**DOI:** 10.1186/s12934-015-0312-7

**Published:** 2015-09-04

**Authors:** Ceren Alkim, Yvan Cam, Debora Trichez, Clément Auriol, Lucie Spina, Amélie Vax, François Bartolo, Philippe Besse, Jean Marie François, Thomas Walther

**Affiliations:** INSA, UPS, INP, LISBP, Université de Toulouse, 135 Avenue de Rangueil, 31077 Toulouse, France; UMR792 Ingénierie des Systèmes Biologiques et des Procédés (LISBP), INRA, Toulouse, France; CNRS, UMR5504, Toulouse, France; TWB, 3 rue Ariane, 31520 Ramonville-St. Agnes, France; UMR CNRS 5219, Institut de Mathématiques de Toulouse, INSA, Université de Toulouse, Toulouse, France

**Keywords:** Synthetic metabolic pathway, Ethylene glycol, Xylose, Metabolic engineering, *Escherichia coli*

## Abstract

**Background:**

Ethylene glycol (EG) is a bulk chemical that is mainly used as an anti-freezing agent and a raw material in the synthesis of plastics. Production of commercial EG currently exclusively relies on chemical synthesis using fossil resources. Biochemical production of ethylene glycol from renewable resources may be more sustainable.

**Results:**

Herein, a synthetic pathway is described that produces EG in *Escherichia coli* through the action of (d)-xylose isomerase, (d)-xylulose-1-kinase, (d)-xylulose-1-phosphate aldolase, and glycolaldehyde reductase. These reactions were successively catalyzed by the endogenous xylose isomerase (XylA), the heterologously expressed human hexokinase (Khk-C) and aldolase (Aldo-B), and an endogenous glycolaldehyde reductase activity, respectively, which we showed to be encoded by *yqhD*. The production strain was optimized by deleting the genes encoding for (d)-xylulose-5 kinase (*xylB*) and glycolaldehyde dehydrogenase (*aldA*), and by overexpressing the candidate glycolaldehyde reductases YqhD, GldA, and FucO. The strain overproducing FucO was the best EG producer reaching a molar yield of 0.94 in shake flasks, and accumulating 20 g/L EG with a molar yield and productivity of 0.91 and 0.37 g/(L.h), respectively, in a controlled bioreactor under aerobic conditions.

**Conclusions:**

We have demonstrated the feasibility to produce EG from (d)-xylose via a synthetic pathway in *E. coli* at approximately 90 % of the theoretical yield.

**Electronic supplementary material:**

The online version of this article (doi:10.1186/s12934-015-0312-7) contains supplementary material, which is available to authorized users.

## Background

Ethylene glycol (EG; 1,2-ethanediol) and its polymers are used in an expanding range of products that include heat transfer fluids, lubricants, surfactants, explosives, cosmetics and plastics [1–3]. The global demand of EG was approximately 21 million tons in 2010 and it is expected to be more than 28 million tons per year by 2015 [4]. Currently, industrial production of EG exclusively relies on chemical synthesis which proceeds through steam cracking of petrol to obtain ethylene, and the oxidation of ethylene to ethylene oxide which is followed by thermal hydrolysis to yield EG [5]. The decreasing availability of petrol and increasing market prices have prompted the search for biochemical production processes to synthesize value-added chemicals from renewable resources [6, 7]. In this context, the microbial production of EG receives increasing attention, and the present study investigates the potential to apply a previously developed synthetic pathway for xylose assimilation [8] for the production of EG.

Three different strategies for the biochemical production of EG from (d)-xylose using engineered *Escherichia coli* strains have recently been described by Liu et al. [9], Stephanopoulos et al. [10], and our group [8] (Table 1), all of which provide access to EG at a theoretical maximum yield of 1 mol/mol. The pathway developed by Liu et al. [9] (in the following termed xylonate pathway) proceeds via the oxidation of (d)-xylose to (d)-xylonate, the dehydration of (d)-xylonate to produce 2-dehydro-3-deoxy-d-pentonate, the aldolytic cleavage of 2-dehydro-3-deoxy-d-pentonate to form pyruvate and glycolaldehyde, and the reduction of the latter to obtain EG. An *E. coli* strain which was deleted in (d)-xylose isomerase, encoded by *xylA*, and which expressed this pathway produced 11.7 g/L EG at a yield of 0.29 g EG per gram (d)-xylose (0.71 mol/mol) in a controlled bioreactor (Table 1). Stephanopoulos et al. [10] developed a pathway [in the following termed (D)-ribulose-1P pathway] that converts (d)-xylose into (D)-ribulose-1P via the sequential action of (d)-xylose isomerase, (d)-xylulose epimerase, and (D)-ribulose-1 kinase. (D)-ribulose-1P is then cleaved aldolytically into dihydroxyacetone phosphate (DHAP) and glycolaldehyde before the latter is reduced to yield EG. A genetically engineered *E. coli* strain expressing this pathway produced 3.5 g/L of EG in shake flasks with a yield of 0.35 g EG per gram xylose consumed (0.84 mol/mol, Table 1). In a controlled bioreactor the strain produced 42 g/L ethylene glycol from an unknown amount of (d)-xylose [10].

**Table 1:** Ethylene glycol production with different engineered *E. coli* strains expressing the synthetic pathway

Experimental conditions	Final conc. (g/L)	Yield^a^ (mol/mol) (g/g)		Productivity [g/(l h)]	References
Xylonate pathway expressed in *E. coli* *ΔxylA* mutant					[9]
Shake flask (MM + 4 g/L xylose + 1 g/L peptone + 0.5 g/L yeast extract)	0.92	0.55	0.23	ni	
Bioreactor (MM + 40 g/L xylose + 10 g/L peptone + 5 g/L yeast extract, batch process)	11.7	0.71	0.29	0.24	
(D)-ribulose-1P pathway (*dte* + *fucA* + *fucO* + *fucK*) expressed in *E. coli* *ΔxylB ΔaldA* (*ΔendA ΔrecA*) mutant					[10]
Shake flask (MM + 10 g/L xylose)	3.5	0.84	0.35	ni	
Bioreactor (MM + unknown amount of xylose, fed-batch process)	42.0	ni	ni	0.6	
(d)-xylulose-1P pathway (*khkC* + *aldoB*) expressed in *E. coli ΔxylB* mutant					[8]
Shake flask (MM + 10 g/L xylose)	1.9	0.45	0.19	ni	
(d)-xylulose-1P pathway (*khkC* + *aldoB* + *fucO*) expressed in *E. coli ΔxylB ΔaldA* mutant					This study
Shake flask (MM + 10 g/L xylose)	4.1	0.94	0.39	ni	
Bioreactor (MM + 55 g/L xylose + 1 g/L peptone, batch process)	20.3	0.91	0.38	0.37	

*MM* mineral salt medium, *ni* not informed.

^a^All pathways listed in this table have a theoretical maximum EG yield on xylose of 0.41 g/g (1 mol/mol).

**Fig. 1:**
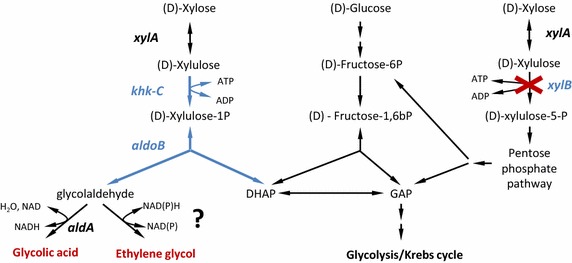
Synthetic (*blue*) and natural (*black*) d-xylose assimilation pathways. In the synthetic pathway (d)-xylose is transformed to (d)-xylulose by endogenous xylose isomerase (XylA). (d)-xylulose-1-kinase (Khk-C) phosphorylates (d)-xylulose to obtain (d)-xylulose-1P, and (d)-xylulose-1-phosphate aldolase (Aldo-B) cleaves (d)-xylulose-1P into glycolaldehyde and DHAP. Ethylene glycol is produced via the action of an unknown endogenous aldehyde reductase.

We recently developed a synthetic pathway [Fig. 1, in the following termed (d)-xylulose-1P pathway] that can transform (d)-xylose into EG and glycolic acid (GA) [8] by proceeding via the isomerization of (d)-xylose to (d)-xylulose, phosphorylation of (d)-xylulose to obtain (d)-xylulose-1P, aldolytic cleavage of (d)-xylulose-1P to form DHAP and glycolaldehyde, and reduction or oxidation of glycolaldehyde to yield EG or GA, respectively. Growth of a strain deleted in the xylulose-5 kinase encoding *xylB* gene on (d)-xylose was restored when (d)-xylulose-1 kinase and (d)-xylulose-1P aldolase, provided by the human genes encoding ketohexokinase-C and aldolase-B enzymes were expressed simultaneously. This strain was then further optimized and eventually produced GA at a molar yield of 0.9 [8].

We herein investigated the potential of our synthetic pathway to produce EG at high yield. We identified the major glycolaldehyde reductase in *E.coli*, and optimized the strain by deleting the endogenous glycolaldehyde oxidase and overexpressing different candidate glycolaldehyde reductases that varied in regard to their cofactor specificities. We then analyzed the impact of different aeration conditions on the production of EG, and found that fully aerobic conditions were preferable over oxygen-limiting and anaerobic conditions. This strategy led us to obtain an engineered strain that produced 20 g/L EG at a molar yield and productivity of approximately 0.9 and 0.3 g/(L h), respectively, in a controlled bioreactor.

## Results and discussion

### The aldehyde reductase YqhD is the major glycolaldehyde reductase in *E. coli*

**Fig. 2:**
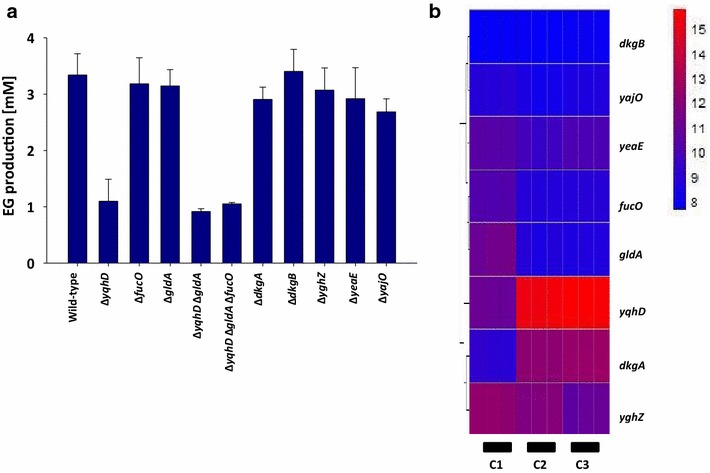
Identification of YqhD as the major glycolaldehyde reductase in *E. coli*. **a** Production of ethylene glycol by *E. coli* strains depending on the deletion of candidate glycolaldehyde reductases. Cells were cultivated on mineral (d)-xylose medium and exposed to 10 mM glycolaldehyde. Production of ethylene glycol was estimated after 10 h of incubation. **b** Log2 transformed expression levels of candidate glycolaldehyde reductases in wild-type cells (C1), strain Pen205 (Δ*xylB* expressing pEXT20-khk-C-aldoB) (C2), and wild-type cells exposed to 10 mM glycolaldehyde (C3). Genes were clustered according to the Euclidean distance between their expression levels using complete-linkage clustering [30]. *Red* and *blue* correspond to high and low expression levels, respectively, using arbitrary units.

We have recently shown that an *E. coli* strain deleted in *xylB* and expressing the synthetic pathway enzymes xylulose-1 kinase and xylulose-1P aldolase, provided by expression of human *khk*-*C* and *aldoB* genes, respectively, was able to grow on xylose [8]. The strain produced EG at a yield of 0.19 g/g (0.45 mol/mol, Table 1). To optimize this strain for EG production we first set out to identify the natural glycolaldehyde reductase in *E. coli.* It was earlier shown that the aldehyde reductases encoded by *dkgA* (*yqhE*), *dkgB* (*yafB*), *yqhD*, *yeaE*, and *yghZ* have glycolaldehyde reductase activity [11]. In addition, the l-1,2-propanediol oxidoreductase encoded by *fucO* [12–14], and the glycerol dehydrogenase encoded by *gldA* [15] were reported to reduce glycolaldehyde [16, 17]. We therefore tested the impact of deleting these enzymes individually or in combination on the capability of *E. coli* strains to reduce glycolaldehyde to EG. The strains carrying the desired mutations were cultivated in shake flasks on minimal medium containing (d)-xylose as the only carbon source. Glycolaldehyde was added at a concentration of 10 mM in the early exponential growth phase (at OD ~1) and the accumulation of EG was measured by HPLC after 10 h of incubation (Fig. 2a). The wild-type strain produced 3.3 mM EG under these conditions. We found that only the deletion of *yqhD* significantly decreased the conversion of glycolaldehyde to EG to approximately 30 % of the wild-type levels. No further decrease of EG production could be observed after additional deletion of *fucO* and *gldA*, which had been employed as glycolaldehyde reductases elsewhere [16, 17] (Fig. 2a). Our results indicate that YqhD functions as the major glycolaldehyde reductase in *E. coli*. This result is somewhat surprising when regarding the comparatively high K_m_ value (28 mM) of YqhD for glycolaldehyde [11]. It is, however, in agreement with the general role of YqhD as a promiscuous aldehyde reductase and detoxifying enzyme in *E. coli* [18]. Moreover, our data indicates that there is at least one additional enzyme capable of reducing glycolaldehyde in the absence of *yqhD*, but we did not identify this enzyme in our study.

We then investigated the transcriptional regulation of the above listed aldehyde reductases in strain Pen205 (*ΔxylB* mutant expressing the synthetic pathway enzymes Khk-C and Aldo-B), and in wild-type cells exposed to 10 mM glycolaldehyde. We re-analyzed our recently published data on the genome-wide transcriptional activity under these conditions [11] and found that among the above listed eight aldehyde reductase-encoding genes only *yqhD* and *dkgA* were induced in strain Pen205 and in the presence of glycolaldehyde (Fig. 2b; Additional file 1: Table S1). The *dkgA* gene was eight- and tenfold induced in strain Pen205 and in wild type incubated with 10 mM glycolaldehyde, respectively, whereas *yqhD* showed the strongest transcriptional upregulation of ~20-fold in strain Pen205 and 26-fold in the presence of glycolaldehyde. The induction of both *yqhD* and *dkgA* could be explained by their regulation via the same transcriptional regulator, YqhC [19], whose expression was increased by sevenfold in strain Pen205 and 15-fold in wild types strain incubated glycolaldehyde.

**Fig. 3:**
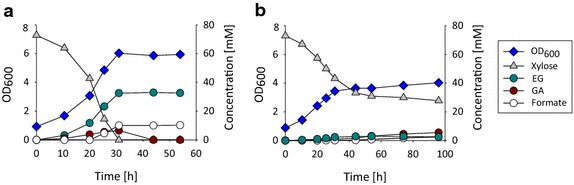
Growth and product formation kinetics depending on the presence and absence of YqhD. **a** Pen205 (Δ*xylB* expressing pEXT20-khk-C-aldoB) and **b** Pen334 (Δ*xylB* Δ*yqhD* pEXT20-khk-C-aldoB) were cultivated on mineral medium containing (d)-xylose at 70 mmol/L initial concentration. Experiments were performed in 250 mL shake flask containing 50 mL medium.

We then analyzed the impact of deleting *yqhD* on growth and EG production of cells expressing the synthetic pathway. Strain Pen334 (*ΔxylBΔyqhD* double mutant expressing the synthetic pathway) was incubated in shake flasks on mineral medium containing (d)-xylose as the sole carbon source. Growth and products formation were monitored by measuring OD_600_ and by carrying out HPLC analyses, respectively. We found that only very small amounts of EG (2.93 ± 0.23 mM) were produced by the *yqhD*-deleted strain Pen334, whereas strain Pen205 accumulated 31 mM EG (Fig. 3). In addition, strain Pen334 consumed only half of the 70 mM (d)-xylose that were initially present in the medium and was unable to sustain growth above 4 units OD_600_, which suggests that the deletion of *yqhD* had caused the accumulation of toxic levels of glycolaldehyde or a derivative of this compound. Taken together, these data indicate that YqhD is the major glycolaldehyde reductase in *E. coli*, and suggest that this enzyme is induced in the presence of the synthetic pathway to detoxify glycolaldehyde to EG.

### Metabolic engineering to increase production of ethylene glycol

As indicated in Table 2, the sole expression of Khk-C and Aldo-B in the wild-type *E. coli* strain (Pen877) did not allow the production EG or GA (Table 2). This result suggested that our synthetic pathway was outcompeted by the natural pathway which channels xylose-derived carbon through the pentose phosphate pathway. Consistently, we showed that the deletion of the xylulose-5 kinase encoded by *xylB* (strain Pen205) was found to be mandatory to enable significant carbon flow and thus production of EG and GA through the synthetic pathway.

**Table 2:** Product yields (Y) of engineered *E. coli* strains expressing the synthetic pathway

Strain name	Deletions	Plasmids	Y_Biomass_ (g/g)	Y_EG_ (mol/mol)	Y_GA_ (mol/mol)	Y_Acetate_ (mol/mol)	Y_Formate_ (mol/mol)
Pen877		pEXT20-khkC-aldoB	0.11 ± 0.00	0.00 ± 0.00	0.00 ± 0.00	1.07 ± 0.02	0.20 ± 0.01
Pen205	*ΔxylB*	pEXT20-khkC-aldoB	0.14 ± 0.00	0.45 ± 0.01	0.09 ± 0.00	0.00 ± 0.00	0.14 ± 0.01
Pen885	*ΔxylB*	pEXT20-khkC-aldoB + pACT3-empty	0.14 ± 0.00	0.33 ± 0.01	0.00 ± 0.00	0.00 ± 0.00	0.17 ± 0.01
Pen222	*ΔxylB*	pEXT20-khkC-aldoB + pACT3-gldA	0.15 ± 0.01	0.37 ± 0.00	0.00 ± 0.00	0.01 ± 0.00	0.13 ± 0.03
Pen223	*ΔxylB*	pEXT20-khkC-aldoB + pACT3-yqhD	0.18 ± 0.03	0.33 ± 0.00	0.03 ± 0.03	0.00 ± 0.00	0.22 ± 0.00
Pen644	*ΔxylB*	pEXT20-khkC-aldoB + pACT3-fucO	0.14 ± 0.01	0.36 ± 0.01	0.06 ± 0.00	0.01 ± 0.00	0.18 ± 0.00
Pen334	*ΔxylB* *ΔyqhD*	pEXT20-khkC-aldoB	0.14 ± 0.00	0.07 ± 0.01	0.04 ± 0.00	0.02 ± 0.00	0.01 ± 0.00
Pen325	*ΔxylB* *ΔaldA*	pEXT20-khkC-aldoB	0.12 ± 0.01	0.88 ± 0.00	0.00 ± 0.00	0.00 ± 0.00	0.02 ± 0.02
Pen361	*ΔxylB* *ΔaldA ΔglcD*	pEXT20-khkC-aldoB	0.10 ± 0.00	0.87 ± 0.00	0.02 ± 0.00	0.00 ± 0.00	0.02 ± 0.00
Pen332	*ΔxylB ΔaldA*	pEXT20-khkC-aldoB + pACT3-gldA	0.08 ± 0.00	0.52 ± 0.00	0.00 ± 0.00	0.00 ± 0.00	0.00 ± 0.00
Pen333	*ΔxylB ΔaldA*	pEXT20-khkC-aldoB + pACT3-yqhD	0.11 ± 0.01	0.90 ± 0.00	0.00 ± 0.00	0.00 ± 0.01	0.01 ± 0.00
Pen641	*ΔxylB ΔaldA*	pEXT20-khkC-aldoB + pACT3-fucO	0.16 ± 0.01	0.94 ± 0.00	0.00 ± 0.00	0.00 ± 0.01	0.07 ± 0.01

Data is presented as means ± standard deviations of at least two independent experiments. For strains Pen334 and 332 only ~50 % of the initially present (d)-xylose were consumed. All experiments were performed in 250 mL shake flasks filled with 50 mL medium and shaken at 200 rpm.

We next tried to increase EG production by overexpressing the major glycolaldehyde reductase YqhD. The gene was cloned into the medium-copy plasmid pACT3 and co-expressed with the synthetic pathway in a *ΔxylB* mutant strain (Pen223). Contrary to expectation, we found that the overproduction of this enzyme caused a drop of the EG yield from 0.45 to 0.33 mol/mol (Table 2). Since an almost identical decrease in EG production was observed for strain Pen885 that carried the empty pACT3 plasmid (Table 2), we concluded that the slight decrease in EG production was likely due the increased metabolic burden caused by the additional plasmid. We then hypothesized that the overexpression of *yqhD* had no positive effect because cofactor supply for this NADPH-dependent enzyme had been limited. We therefore tested the impact of expressing the NADH-dependent enzymes FucO and GldA which were previously shown to have glycolaldehyde reductase activity [16, 17]. As shown in Table 2, the expression of those enzymes also resulted in a decrease of EG production that was comparable to what had been observed for the overproduction of YqhD.

These results suggested that the glycolaldehyde reductases were outcompeted by the glycolaldehyde dehydrogenase(s) which oxidizes glycolaldehyde to glycolate (Fig. 1). We have previously shown that overexpression of the short chain aldehyde dehydrogenase, AldA [20] abolished EG production in a strain that expressed our synthetic pathway [8]. We therefore tested the effect of deleting this gene on EG production. We found that the *aldA*-deleted strain (Pen325) produced EG at a molar yield of 0.88 which corresponded to an increase of nearly 100 % compared to strain Pen205. This result showed that AldA plays a major role in the assimilation of the glycolaldehyde that is released by our synthetic pathway. It is of interest to note that the observed positive effect of deleting *aldA* is at variance with data of Liu et al. [9] who found that the *aldA* deletion was toxic for the cells. It, however, agrees with Stephanopoulos et al. [10] who proposed to delete *aldA* to increased EG production.

The theoretical C2-compound yield (Y_C2_ = ([GA] + [EG])/[xylose_consumed_]) of our pathway is 1 mol ([EG] + [GA]) per mol xylose (Fig. 1). Thus, despite the increase of EG production to 88 % of the theoretical yield in strain Pen325, part of the glycolaldehyde was still consumed by another pathway. We therefore tested whether an additional glycolaldehyde dehydrogenase was active in our strains which could explain the incomplete recovery of the glycolaldehyde-derived carbon fraction. The inactivation of glycolate oxidase, encoded by *glcDEFG* [21], results in the incapacity of *E. coli* to consume GA [11, 22]. We therefore deleted the glycolate oxidase subunit *glcD* in addition to *aldA* to obtain strain Pen361 (*ΔxylB ΔaldA ΔglcD* + pEXT20-*khkC*-*aldoB*) and tested whether we could detect GA in the supernatant of this strain which would be an indicator for the presence of another unknown glycolaldehyde dehydrogenase. We observed that strain Pen361 produced only trace amounts of GA (0.02 mol/mol, Table 2). This result showed that the apparent ~12 % loss of glycolaldehyde observed for strain Pen325 did not occur through the glycolate/glyoxylate pathway. We therefore conclude that a small fraction of glycolaldehyde was consumed by an unknown metabolic pathway which remains to be identified.

A further incremental improvement of EG production to molar yields of 0.9 and 0.94 was achieved when the glycolaldehyde reductases YqhD or FucO, respectively, were overexpressed in the strain carrying the *aldA* deletion. In contrast to the positive impact of YqhD and FucO, the overexpression of GldA only had a slightly negative effect (Table 2). YqhD and FucO were earlier applied as glycolaldehyde reductases [9, 10]. We here compared the effect of expressing either of these enzymes and found that both had a small positive effect on EG production.

### Optimization of aeration conditions to increase production of ethylene glycol

After showing that the deletion of *aldA* encoding the glycolaldehyde dehydrogenase and the overexpression of *yqhD* or *fucO* encoding glycolaldehyde reductases increased the production of EG, we investigated which aeration conditions are best for EG production by our synthetic pathway. We hypothesized that decreasing aeration of the cultures may increase the supply of reducing cofactors in particular for the NADH-dependent glycolaldehyde reductases GldA and FucO.

We varied the oxygen supply to our cultures by incubating them in 250 mL shake flasks which were filled with 50 mL (condition 1, as above) or 100 mL (condition 2) of culture medium, thus imposing gradually decreasing aeration with increasing medium volume [23]. In addition, anaerobic cultivation conditions (condition 3) were analyzed by cultivating the cells in completely filled 100 mL glass bottles that were sealed with a rubber cap. The anaerobic bottles were inoculated aerobically and anaerobic conditions were attained after only few minutes of cultivation due the oxygen consumption of the growing culture. Absence of oxygen was verified by the characteristic pink color of the redox indicator resazurin [24].

We tested the impact of these different aeration conditions on *ΔxylB ΔaldA* double mutant strain Pen325 which only expressed the synthetic pathway that comprised of Khk-C and Aldo-B, or the synthetic pathway in combination with one of the three glycolaldehyde reductases GldA (Pen332), YqhD (Pen333), or FucO (Pen641). The results are summarized in Table 3. We found that fully aerobic conditions (condition 1) were preferable over micro-aerobic (condition 2) and anaerobic conditions (condition 3) for the production of EG when using the strains that overproduced YqhD (Pen333), FucO (Pen641), or which contained no additional glycolaldehyde reductase (Pen325). Upon decreasing oxygen supply and in the absence of oxygen these strains accumulated increasing amounts of acetate and succinate in the supernatant, which negatively affected biomass and EG yield. Curiously we could detect up to 0.17 mol/mol formate (Pen641) in the supernatant of these cultures under micro-aerobic conditions but not under anaerobic conditions. A slight exception of this preference for fully aerobic conditions was strain Pen332 which expressed GldA. These cells exhibited a significantly increased production of EG of 0.74 mol/mol under micro-aerobic conditions as compared to only 0.52 mol/mol under fully aerobic conditions.

**Table 3:** The impact of variations in oxygen supply on the production of ethylene glycol

Strain name	Genotype and plasmids	Culture condition	Y_Biomass_ (g/g)	Y_EG_ (mol/mol)	Y_Acetate_ (mol/mol)	Y_Formate_ (mol/mol)	Y_Succinate_ (mol/mol)
Pen325	*ΔxylB ΔaldA* pEXT20-khkC-aldoB	1	0.12 ± 0.01	0.88 ± 0.01	0.00 ± 0.00	0.02 ± 0.02	0.00 ± 0.00
		2	0.09 ± 0.00	0.87 ± 0.00	0.05 ± 0.01	0.13 ± 0.00	0.00 ± 0.00
		3	0.06 ± 0.01	0.65 ± 0.00	0.70 ± 0.18	0.00 ± 0.00	0.00 ± 0.00
Pen332	*ΔxylB ΔaldA* pEXT20-khkC-aldoB pACT3-gldA	1	0.08 ± 0.01	0.52 ± 0.02	0.00 ± 0.00	0.00 ± 0.00	0.00 ± 0.00
		2	0.08 ± 0.02	0.74 ± 0.05	0.01 ± 0.00	0.04 ± 0.00	0.00 ± 0.00
		3	0.04 ± 0.01	0.46 ± 0.03	0.30 ± 0.12	0.01 ± 0.00	0.19 ± 0.02
Pen333	*ΔxylB ΔaldA* pEXT20-khkC-aldoB pACT3-yqhD	1	0.11 ± 0.01	0.90 ± 0.01	0.00 ± 0.01	0.01 ± 0.00	0.00 ± 0.00
		2	0.08 ± 0.00	0.79 ± 0.04	0.10 ± 0.14	0.11 ± 0.03	0.00 ± 0.00
		3	0.03 ± 0.01	0.59 ± 0.10	0.50 ± 0.12	0.01 ± 0.00	0.17 ± 0.02
Pen641	*ΔxylB ΔaldA* pEXT20-khkC-aldoB pACT3-fucO	1	0.16 ± 0.01	0.94 ± 0.00	0.00 ± 0.00	0.07 ± 0.01	0.00 ± 0.00
		2	0.08 ± 0.01	0.80 ± 0.00	0.18 ± 0.06	0.17 ± 0.08	0.00 ± 0.00
		3	0.05 ± 0.00	0.61 ± 0.12	0.42 ± 0.11	0.01 ± 0.00	0.20 ± 0.07

(1) 50 mL medium in 250 mL shake flasks, 200 rpm. (2) 100 mL medium in 250 mL shake flasks, 100 rpm. (3) anaerobic cultures, 200 rpm. Data is presented as means ± standard deviations of at least two independent experiments.

These results indicate that (1) fully aerobic conditions are required for the production of EG at high yield, and (2) further support the hypothesis that an unknown glycolaldehyde-consuming pathway must be present in *E. coli*, because the recovery of the C2 carbon fraction (in the form of EG or GA) dropped to only ~0.5–0.6 mol per mol xylose under anaerobic conditions.

### Production of ethylene glycol in controlled bioreactors

**Fig. 4:**
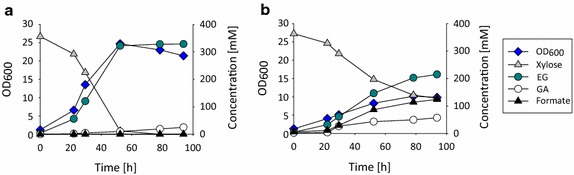
Growth and product formation kinetics of strain Pen641 in controlled bioreactors. Cells were cultivated under fully aerobic conditions (**a**), or under micro-aerobic conditions (**b**) on xylose mineral medium enriched with 1 g/L tryptone and 0.5 g/L yeast extract. Initial (d)-xylose concentration was 55 g/L. Aerobic and micro-aerobic conditions were imposed by maintaining the dissolved oxygen tension above 40 and 2 %, respectively.

To explore the capacity of strain Pen641 for maximal EG to production, we analyzed growth and products formation of this strain in controlled bioreactors under two different aeration conditions. We imposed fully aerobic conditions in one experiment by maintaining the dissolved oxygen tension above 40 % throughout the cultivation. In parallel, we analyzed the impact of a reduced oxygen supply by allowing the dissolved oxygen tension to decrease to 2 % in another experiment. Strain Pen641 was pre-cultured in (d)-xylose mineral medium until cells grew exponentially. These cells were then used to inoculate 0.5 l bioreactors that contained 250 mL mineral medium with an initial (d)-xylose concentration of 55 g/L supplemented with 1 g/L tryptone and 0.5 g/L yeast extract at an OD of ~1.2. The pH of the cultures was kept at 7.0 by the addition of 10 M KOH. The fermentation kinetics of these experiments are depicted in Fig. 4. Under aerobic conditions strain Pen641 produced 20 g/L EG with a volumetric productivity of 0.37 g/(L h). The molar EG yield on (d)-xylose was 0.9 (0.38 g/g) and only small amounts of the metabolic by-products glycolaldehyde (1 g/L) and formate (0.45 g/L) were produced (Fig. 4a). Under oxygen-limited conditions strain Pen641 produced EG also at a molar yield of 0.9. However, when oxygen supply was limited the strain was not able to consume all the xylose in the medium and accumulated only 13.3 g/L EG with a significantly decreased volumetric productivity of only 0.15 g/(L. h) (Fig. 4b). Under these conditions, cells exhibited a twofold reduced growth rate (0.05/h as compared to 0.1/h under fully aerobic conditions), and they ceased growth at an OD_600_ of ~10 presumably because the acetate concentration of 7 g/L in the medium became growth inhibiting. These data further support the notion that fully aerobic conditions are preferable for EG production when using the described synthetic pathway. The almost complete absence of metabolic by-products and notably acetate under these conditions suggest that it is possible to accumulate EG to significantly higher concentrations in fed-batch fermentation processes.

The EG yield and productivity achieved by our strain are by 27 and 54 %, respectively, higher than for the strain carrying the xylonate pathway engineered by Liu et al. [9]. Given the strong transient accumulation of glycolic acid in the fermentation experiment of Liu et al. [9], the increase of EG yield obtained with our strain can be attributed in large part to the favorable effect of deleting glycolaldehyde dehydrogenase AldA which avoids carbon loss occurring through the glycolate/glyoxylate pathway. In addition, our strain does not accumulate synthetic pathway intermediates, as it is the case for xylonate in the fermentation carried out by Liu et al. [9], which positively impacts the productivity of our synthetic pathway. The strain developed by Stephanopoulos et al. [10] which carried the (D)-ribulose-1P pathway produced 42 g/L EG in a fed-batch process at a rate of 0.6 g/(L h). No information is available on the actual carbon yield of their fermentation process and on the formation of metabolic by-products. However, in shake flasks this strain reached nearly the same EG yield as our strain (Table 1) which suggests that it should be possible to reach similar or even higher yield and productivity through our (D)-xylulose-1P synthetic pathway upon further process optimization.

## Conclusions

We have proposed a synthetic pathway for the assimilation of (d)-xylose which relies on the carbon-conserving aldolytic cleavage of the C5 molecule (d)-xylulose-1P into the C2 molecule glycolaldehyde and the C3 molecule DHAP, thus providing an efficient access to the C2 compounds EG and GA [8]. Spontaneous production of EG upon expression of the synthetic pathway in *E. coli* was shown to depend on the aldehyde reductase YqhD which is induced in the presence of the pathway intermediate glycolaldehyde. The key factors for the optimization of EG production were the deletion of *aldA* encoding the glycolaldehyde dehydrogenase, and the cultivation of the optimized strains under aerobic conditions. While EG cannot be produced from (d)-xylose via the annotated metabolic network in *E. coli*, we achieved a yield of approximately 0.9 mol/mol by applying the new pathway in the genetically optimized production strain. From a conceptual perspective this demonstrates that the stoichiometry of sugar assimilation can be strongly altered such that product formation is favored over cell growth. However, to increase the overall carbon yield of the process, it is required to convert at least part of the DHAP which is released by the synthetic pathway into the desired product. It is therefore of strong interest to test whether the established and suggested metabolic pathways for the conversion of DHAP into GA [25] and EG [10] can be combined with the metabolic pathway developed in our study.

## Methods

### Growth media and culture conditions

Luria–Bertani (LB) medium [26] or M9 minimal medium which contained (d)-glucose or (d)-xylose were used throughout this study. All genetic manipulations were carried out in LB medium. Growth of the cultures was realized in M9 minimal medium that contained (d)-glucose or (d)-xylose at concentrations of 20 or 10 g/L, respectively, together with 18 g/L Na_2_HPO_4_ · 12H_2_O, 3 g/L KH_2_PO_4_, 0.5 g/L NaCl, 2/L g NH_4_Cl, 0.5 g/L MgSO_4_ · 7H_2_O, 0.015 g/L CaCl_2_ · 2H_2_O, 0.010 g/L FeCl_3_, 0.006 g/L Thiamine HCl, 0.4 mg/L NaEDTA · 2H_2_O, 1.8 mg/L CoCl_2_ · 6H_2_O, 1.8 mg/L ZnCl_2_SO_4_ · 7H_2_O, 0.4 mg/L Na_2_MoO_4_ · 2H_2_O, 0.1 mg/L H_3_BO_3_, 1.2 mg/L MnSO_4_ · H_2_O, 1.2 mg/L CuCl_2_ · 2H_2_O. 3-(*N*-morpholino)propanesulfonic acid (MOPS) solution at pH 7 was used to buffer M9 minimal medium to a final concentration of 20 g/L after filter sterilization (Merck Millipore ExpressPlus). The media were filter sterilized or autoclaved. When required, antibiotics were added to the media at a concentration of 100, 50, and 25 µg/mL for ampicillin, kanamycin and chloramphenicol, respectively. Isopropyl *β*-d-1-thiogalactopyranoside (IPTG) was added to a final concentration of 1 mM when needed. All chemicals were from Sigma-Aldrich.

Growth of the cultures was followed by measuring the optical density at 600 nm (OD_600_) using a spectrophotometer (Biochrom Libra S11). Pre-cultures were grown overnight in 10 mL of LB medium in 50-mL test tubes (BD Falcon) on a rotary shaker (Infors HT) running at 200 rpm at 37 °C. They were harvested by centrifugation (4,000×*g*, Allegra 21-R, Beckman-Coulter), washed once with sterile water and transferred to 250 mL shake flasks containing M9 minimal medium with glucose adjusting an initial OD_600_ of 0.25. IPTG (1 mM) was added to the cultures when the OD_600_ reached ~0.8 to assure proper expression of the synthetic pathway enzymes before transfer to xylose medium. After overnight incubation cells were harvested by centrifugation, washed with sterile water and used to inoculate M9 medium containing xylose and 1 mM IPTG. Different aeration conditions were imposed by adding 50 or 100 mL medium into the 250 mL shake flasks. Anaerobic conditions were imposed by culturing the cells in 100 mL medium shaken in 100 mL culture bottles that were sealed with rubber caps. Absence of oxygen was verified by adding 1 mg/mL of the redox indicator sodium resazurin (Sigma-Aldrich) to the medium. All cultures were incubated at 37 °C and shaken at 200 rpm except for the condition with 100 mL medium in 250 mL flasks which was shaken at 100 rpm to increase oxygen limitation.

### Bioreactor cultures

The pre-cultures for the inoculation of the bioreactors were cultivated in 500 mL shake flasks containing 100 mL xylose mineral medium (composition as indicated above) until exponential phase. The cells were harvested by centrifugation (4,500×*g*, Sorvall STR40) and used to inoculate 0.5 l bioreactors (MiniBio, Applikon) that contained 250 mL medium at an OD_600_ of ~1.2. The composition of the fermentation medium was similar to the mineral medium used in the shake flask experiments, except that it contained 55 g/L (d)-xylose, 2 g/L Na_2_HPO_4_ · 12H_2_O, 0.8 g/L KH_2_PO_4_, 6 g/L (NH_4_)_2_HPO_4_, 0.4 g/L (NH_4_)_2_SO_4_, 1 g/L tryptone (Biokar), 0.5 g/L yeast extract (Biokar), 0.4 mL/L polypropylene glycol as antifoaming agent, 1 mM IPTG, and no MOPS. The pH of the cultures was kept at 7.0 by the addition of 10 M KOH, and reactors were aerated with air at 1 vvm. Dissolved oxygen tension was regulated by adjusting the appropriate agitation speed (300–1,200 rpm, Rushton rotor, 28 mm diameter), and was either kept at 40 % to impose fully aerobic conditions, or at 2 % to impose micro-aerobic conditions.

### Determination of extracellular metabolite concentrations

Sugar consumption and production of EG, AG, and other organic acids was followed by regularly withdrawing samples from the supernatant which were centrifuged at 15,700*g* for 5 min (Eppendorf 5415D), filtered (Sartorius Minisart RC4), and kept at −20 °C before being analyzed by HPLC as described previously [8].

### RNA extraction, microarray analysis, and data treatment

A detailed description of the experimental conditions for RNA extraction and microarray analysis is provided in [8]. Briefly, all experiments were carried on M9 mineral medium containing 10 g/L (d)-xylose as the only carbon source. Wild-type cells, wild-type cells exposed to 10 mM glycolaldehyde, and strain Pen205 were harvested during early exponential phase at an OD_600_ of ~1. Glycolaldehyde was added to the cultures 30 min before harvest. Cells were separated from the culture medium by centrifugation (1,500×*g*, 5 min, Eppendorf 5415D). After discarding the supernatant RNA was extracted from the cells by using the RNeasy Mini Kit (QIAGEN). Quantity and quality of the samples were determined by NanoDrop (Thermo) and Bioanalyzer (Agilent Technologies), respectively. The Low Input Quick Amp Labeling kit (Agilent) was used to convert RNA samples to labeled cDNA which was hybridized on *E. coli* Gene Expression Microarrays (8 × 15 K, Agilent) following the Agilent One-Color Microarray-Based Gene Expression Analysis Protocol. The slides were scanned on a Tecan scanner MS200 and analyzed by Feature Extraction V.11.5.1.1. RNA was extracted and analyzed from three independent experiments for each condition. Background correction and normalization of the transcriptome data was carried out as described previously [27–29].

To produce the heatmap shown in Fig. 2b, the expression levels of the corresponding genes were log2 transformed, and the genes were clustered according to the Euclidean distance between their expression levels using complete-linkage clustering [30].

### Strain and plasmid construction

(Strain construction) *Escherichia coli* K-12 substr. MG1655 (ATCC 47076) was used as the parental strain throughout this study. The deletion mutants were created by the phage transduction method adapted from Miller [31]. Briefly: (Lysate preparation) The phage lysates were prepared from strains of the KEIO collection [32] which carried single deletions. The corresponding deletion mutant was grown in 5 mL of LB medium in 50 mL test tubes (BD Falcon) at 37 °C and 200 rpm overnight. The next day, 200 µL of this pre-culture was inoculated into fresh 10 mL LB medium supplemented with glucose (0.2 %) and CaCl_2_ (5 mM). It was then cultivated for 1 h at 37 °C and 200 rpm. 100 µL of phage lysate P1 was then added into the culture. 200 µL chloroform was added to the culture after 2 h of incubation at 37 °C and 200 rpm. Cells were harvested by centrifugation at 4,500*g* for 10 min (Allegra 21-R, Beckman-Coulter). The supernatant was transferred to a new tube and 200 µL chloroform were added. The lysates were stored at 4 °C until transduction was carried out. (Transduction) 1.5 mL of an overnight culture were resuspended in 10 mM MgSO_4_ and 5 mM CaCl_2_. 100 µL of this suspension was mixed with 100 µL of the lysate and incubated at 30 °C for 30 min. Then 100 µL of 1 M trisodium citrate (Na_3_C_6_H_5_O_7_) solution was added to the cells. They were mixed and 400 µL of LB was added into the cells. They were incubated at 37 °C and 200 rpm for 1 h. Cells were inoculated on LB agar plates that contained kanamycin. Positive clones were verified by PCR analysis. All strains used and constructed in this study are indicated in Table 4.

**Table 4:** *Escherichia coli* strains used in this study

Strain reference	Genotype	References
MG1655	F^−^λ^−^ ilvG-rfb-50 rph-1	ATCC 47076
NEB5-α	*fhuA2 Δ*(*argF*-*lacZ*)*U169 phoA glnV44 Φ80Δ* (*lacZ*)*M15 gyrA96 recA1 relA1 endA1 thi*-*1 hsdR17*	NEB
JW3536-2	F-, *Δ*(*araD*-*araB*)*567*, *ΔlacZ4787*(*::rrnB*-*3*), *λ*-, *ΔxylB747::kan*, *rph*-*1*, *Δ*(*rhaD*-*rhaB*)*568*, *hsdR514*	[32]
JW2978-1	F-, *Δ*(*araD*-*araB*)*567*, *ΔlacZ4787*(::rrnB-3), *λ* ^−^, *ΔyqhD783::kan*, *rph*-*1*,*Δ*(*rhaD*-*rhaB*)*568*, *hsdR514*	[32]
JW1412-1	F-, *Δ*(*araD*-*araB*)*567*, *ΔlacZ4787*(::rrnB-3), *λ* ^−^, *ΔaldA776::kan*, *rph*-*1*, *Δ*(*rhaD*-*rhaB*)*568*, *hsdR514*	[32]
JW1375-1	F-, *Δ*(*araD*-*araB*)*567*, *ΔlacZ4787*(::rrnB-3), *λ* ^−^, *ΔldhA744::kan*, *rph*-*1*, *Δ*(*rhaD*-*rhaB*)*568*, *hsdR514*	[32]
JW2770-1	F-, *Δ*(*araD*-*araB*)*567*, *ΔlacZ4787*(::rrnB-3), *λ* ^−^, *ΔfucO726::kan*, *rph*-*1*,*Δ*(*rhaD*-*rhaB*)*568*, *hsdR514*	[32]
JW5556-3	F-, *Δ*(*araD*-*araB*)*567*, *ΔlacZ4787*(::rrnB-3), *λ* ^−^, *rph*-*1*, *Δ*(*rhaD*-*rhaB*)*568*, *ΔgldA732::kan*, *hsdR514*	[32]
JW5499-1	F-, *Δ*(*araD*-*araB*)*567*, *ΔlacZ4787*(::rrnB-3), *λ* ^−^, *ΔdkgA784::kan*, *rph*-*1*, *Δ*(*rhaD*-*rhaB*)*568*, *hsdR514*	[32]
JW0197-1	F-, *Δ*(*araD*-*araB*)*567*, *ΔdkgB726::kan*, *ΔlacZ4787*(::rrnB-3), *λ* ^−^, *rph*-*1*,*Δ*(*rhaD*-*rhaB*)*568*, *hsdR514*	[32]
JW2970-1	F-, *Δ*(*araD*-*araB*)*567*, *ΔlacZ4787*(::rrnB-3), *λ* ^−^, *ΔyghZ775::kan*, *rph*-*1*,*Δ*(*rhaD*-*rhaB*)*568*, *hsdR514*	[32]
JW1770-5	F-, *Δ*(*araD*-*araB*)*567*, *ΔlacZ4787*(::rrnB-3), *λ* ^−^, *ΔyeaE778::kan*, *rph*-*1*, *Δ*(*rhaD*-*rhaB*)*568*, *hsdR514*	[32]
JW0409-1	F-, *Δ*(*araD*-*araB*)*567*, *ΔlacZ4787*(::rrnB-3), *ΔyajO778::kan*, *λ* ^−^, *rph*-*1*, *Δ*(*rhaD*-*rhaB*)*568*, *hsdR514*	[32]
Pen79	JW2978-1 *ΔgldA::FRT*	This work
Pen99	Pen79 *ΔfucO::FRT*	This work
Pen155	MG1655 *ΔxylB::FRT*	[8]
Pen205	Pen155 containing pEXT20-khkC-aldoB	[8]
Pen222	Pen205 containing pACT3-gldA	This work
Pen223	Pen205 containing pACT3-yqhD	This work
Pen259	Pen155 *ΔyqhD::FRT*	This work
Pen278	Pen155 *ΔaldA::FRT*	This work
Pen325	Pen278 containing pEXT20-khkC-aldoB	This work
Pen332	Pen325 containing pACT3-gldA	This work
Pen333	Pen325 containing pACT3-yqhD	This work
Pen334	Pen205 *ΔyqhD::FRT* containing pEXT20-khkC-aldoB	This work
Pen345	Pen278 *ΔglcD::FRT*	This work
Pen361	Pen345 containing pEXT20-khkC-aldoB	This work
Pen641	Pen325 containing pACT3-fucO	This work
Pen644	Pen205 containing pACT3-fucO	This work
Pen877	MG1655 containing pEXT20-Khk-C-aldoB	This work
Pen885	Pen205 containing empty pACT3	This work

(Plasmid construction) Construction of plasmid pEXT20-khkC-aldoB was described previously [8]. The glycolaldehyde-encoding genes *gldA*, *yqhD* and *fucO* were PCR amplified from *Escherichia coli* K-12 MG1655 genomic DNA using Phusion polymerase (Biolabs) and the corresponding primers listed in Table 5. The PCR products were then purified by using a PCR purification kit (Thermo Scientific). The *In*-*Fusion*^®^ HD Cloning Kit (Clontech) was used to recombine the DNA fragments into the pACT3 expression vector that was digested with *Pst*I/*Hin*dIII. The vectors obtained were named pACT3-gldA, pACT3-yqhD and pACT3-fucO (Table 6).

**Table 5:** Primers used in this study

Primer	Sequence
Cloning of gldA	
gldA_rbs_f	CCTCTAGAGTCGAC*CTGCAG*AGGAGGATTCAT***ATG***GACCGCATTA
gldA_rbs_r	GCCAAAACAG*AAGCTT*TTATTCCCACTCTTGCAGG
Cloning of fucO	
fucO_rbs_f	TT*GGATCC*AGGAGGATTCAT***ATG***GCTAACAGAATGATTCT
fucO_rbs_r	TT*AAGCTT*TTACCAGGCGGTATGGTAA
Cloning of yqhD	
yqhD_rbs_f	CCTCTAGAGTCGAC*CTGCAG*AGGAGGATTCAT***ATG***AACAACTTTA
yqhD_rbs_r	GCCAAAACAG*AAGCTT*TTAGCGGGCGGCTTCGT
Verification primers for gene knock-outs	
xylB_loc_f	GTTATCGGTAGCGATACCGGGCATTTT
xylB_loc_r	GGATCCTGAATTATCCCCCACCCGGTCAGGCA
yqhD_loc_f	CGCCATACAACAAACGCACA
yqhD_loc_r	CCAGATGCCAGCGGATAACA
gldA_loc_f	CGGTTCAGGAGCTGCAAACGCTG
gldA_loc_r	TAAGAGTCACAGATTCGACCTTC
fucO_loc_f	ACAACATCATGGGCTTATCG
KANseq_rev	ATGCGATGTTTCGCTTGGTG
aldA_loc_f	TCATCCATGCATGGCAAACG
aldA_loc_r	ACTGCCGAAGAGGTGAATAA

Restriction sites are italicized and the start codons are shown in bolditalics.

**Table 6:** Plasmids used in this study

Name	Relevant characteristics	References
pGEM-T	Amp^R^, used for PCR fragment subcloning	Promega
pACT3	Cm^R^	[33]
pEXT20	Amp^R^	[33]
pCP20	Amp^R^, plasmid used for removing Kan cassette	[34]
pEXT20-khkC-aldoB	pEXT20 derivative carrying both *H. sapiens* *khk*-*c* gene from Asipu et al., 2003 and *aldoB* gene	[8]
pACT3-gldA	pACT3 derivative carrying *gldA* gene from *E. coli*	This work
pACT3-yqhD	pACT3 derivative carrying *yqhD* gene from *E. coli*	This work
pACT3-fucO	pACT3 derivative carrying *fucO* gene from *E. coli*	This work

## Additional file

Additional file 1. Log2 transformed expression levels of candidate glycolaldehyde reductases
